# Can 3D-Printed Bioactive Glasses Be the Future of Bone Tissue Engineering?

**DOI:** 10.3390/polym14081627

**Published:** 2022-04-18

**Authors:** Amey Dukle, Dhanashree Murugan, Arputharaj Joseph Nathanael, Loganathan Rangasamy, Tae-Hwan Oh

**Affiliations:** 1 Centre for Biomaterials, Cellular and Molecular Theranostics (CBCMT), Vellore Institute of Technology (VIT), Vellore 632014, Tamil Nadu, India; amey.dukle@vit.ac.in (A.D.); dhanashree.murugan2019@vitstudent.ac.in (D.M.); loganathan.r@vit.ac.in (L.R.); 2 School of Biosciences & Technology (SBST), Vellore Institute of Technology (VIT), Vellore 632014, Tamil Nadu, India; 3 School of Chemical Engineering, Yeungnam University, Gyeongsan 38541, Korea

**Keywords:** bioactive glass, bioglass, composite, scaffold, tissue engineering, bioprinting, polymer

## Abstract

According to the Global Burden of Diseases, Injuries, and Risk Factors Study, cases of bone fracture or injury have increased to 33.4% in the past two decades. Bone-related injuries affect both physical and mental health and increase the morbidity rate. Biopolymers, metals, ceramics, and various biomaterials have been used to synthesize bone implants. Among these, bioactive glasses are one of the most biomimetic materials for human bones. They provide good mechanical properties, biocompatibility, and osteointegrative properties. Owing to these properties, various composites of bioactive glasses have been FDA-approved for diverse bone-related and other applications. However, bone defects and bone injuries require customized designs and replacements. Thus, the three-dimensional (3D) printing of bioactive glass composites has the potential to provide customized bone implants. This review highlights the bottlenecks in 3D printing bioactive glass and provides an overview of different types of 3D printing methods for bioactive glass. Furthermore, this review discusses synthetic and natural bioactive glass composites. This review aims to provide information on bioactive glass biomaterials and their potential in bone tissue engineering.

## 1. Introduction

Bone transplants are one of the most widely performed tissue transplant procedures, second only to blood transfusion [1,2]. The main reasons for bone transplants are bone disorders caused by various factors, such as birth defects, accidents, trauma, tumors, and infections (e.g., osteomyelitis). In most cases, bone injuries heal independently owing to the immune response of the body [3]. However, external intervention in the form of bone grafts may be required in special cases. Bone grafts are used for the treatment of bone defects that are unable to heal on their own owing to slow self-healing in the body or large defect sizes [4].

Bone grafts are typically required when the size of the defect is 2–2.5-times larger than the diameter of the bone [5]. These bone grafts are instrumental in the sequential healing process, which includes inflammation of the affected site, osteogenesis and resorption phases, revascularization, and eventually their incorporation in the host skeleton [6,7].

Bone grafts can be sourced from the patient’s body (autografts), donors from the same source species (allografts), and donors from different species (xenografts) [8,9]. Among the three types of bone grafts, xenografts have not been widely used due to a lack of reliable results in clinical studies [10]. Allografts from other individuals pose a risk of disease transmission and patient body rejection [11,12]. Autografts have a low chance of rejection in the recipient’s body. However, available donor sites in the patient’s body are limited, which limits the amount of bone that can be harvested.

In addition, there are reports of inflammation occurring at the donor site [13]. For these reasons, there is a demand for a substitute material that can fulfill the functions of bone grafts and is freely available [12,14,15,16]. This has led to the progression of bone tissue engineering as a promising industry.

In bone tissue engineering, artificial materials are developed as substitutes for bone grafts for bone healing and regeneration processes [17,18]. The artificial scaffold used is implanted to support the defect site and assist in the healing process [19]. During the surgical procedure, the defect site is cleaned, and the scaffold is surgically implanted at the site [17]. The scaffold provides an artificial extracellular matrix for the attachment of osteoblast stem cells and allows their proliferation to complete the healing process [20]. An ideal bone tissue engineering scaffold should have the following properties/functionalities:

- Biocompatible: a scaffold should perform the function of cell recruitment and offer a conducive surface for proliferation. It should be porous to provide a large surface area for cell attachment and proliferation.
- Biodegradable: a scaffold should dissolve completely when bone regeneration is complete. Ideally, the rate of scaffold degradation should match the rate of new bone formation.
- Biomimetic: the human bone is a hierarchical structure comprising a ring-type matrix [21,22,23,24]. An ideal scaffold mimics the bone in terms of its hierarchical structure. The mechanical properties of human bones are highly anisotropic. An elastic modulus of cortical bone is 11,500 MPa in the transverse direction and 17,000 MPa in the longitudinal direction. The trabecular bone is 400 MPa longitudinally [25]. The mechanical properties of an ideal scaffold should match the mechanical properties of the bone it is used in.
- Bio-functional: a scaffold should accelerate the healing process by delivering drugs and promoting growth factors to the defect site.

Numerous materials, such as metals [26], polymers [27], ceramics [20,28,29,30,31], and composites [32,33], have been studied for scaffold production. First-generation materials used for scaffold preparation, including traditional materials, such as metals and polymers, are bioinert [34]. These scaffolds do not undergo any degradation when implanted into the body.

Moreover, scaffolds made of certain materials are encapsulated by fibrous tissues and thus separated from the functioning of the body [10,19]. All these properties have led to a demand for second-generation bioactive materials [34]. These materials undergo active interaction with the body, accelerate the healing process and deliver drugs and growth factors. Prominent examples of bioactive materials are ceramics, such as hydroxyapatite, dicalcium phosphate, β-tricalcium phosphate, amorphous calcium phosphate, and bioactive glasses [35].

The human bone is mainly composed of hydroxyapatite and collagen type I, along with mi-nor components, such as non-collagenous proteins and lipids [36]. The protein components of the bone provide tensile strength to the bone, whereas the mineral components of the bone—for instance, hydroxyapatite—provide compressive strength [37]. This naturally makes hydroxyapatite a favorite material of bone tissue engineers. Hydroxyapatite and its composites display sufficient mechanical strength and osteoconductive behavior [38]. Hydroxyapatite and its composites have been widely studied by various research groups and in clinical studies [38,39,40,41].

Bioactive glass is an equally promising material but less widely explored. Bioactive glass implanted into the body forms an amorphous calcium phosphate or crystalline hydroxyapatite phases on its surface, forming strong bonds with bone tissue [34,42]. Bioactive glass provides highly reactive surface area; the hydroxycarbonated apatite layer enhances protein adsorption on the surface of the implants [43].

These initial reactions in the apatite layer increase ion release from the bioactive glass surface. This ion generation was found to enhance biocompatibility by promoting osteoconductivity [44,45].

As of these properties, bioactive glass is FDA-approved and is commercially available as implants, including the 45S5 Bioglass^®^ implant (for middle ear ossicular repair), PerioGlas (for bone and dental repair), TheraSphere^®^ (for cancer treatment), and Medpor^®^-Plus™ (for porous orbital implants) [44]. It can also be used as a drug delivery vehicle for therapeutic ions and growth factors. However, pure bioactive glass cannot be used for scaffold preparation because it loses its amorphous properties at high sintering temperatures. Polymer-bioactive glass composites have thus been widely explored as a scaffold material. Composite scaffolds utilize the biofunctionality, biocompatibility, and biodegradability of bioactive glass along with the mechanical properties of polymers to develop a superior biomimetic scaffold.

To develop scaffold materials for bone tissue engineering, a synergy is required between scientists and surgeons. An engineer may prioritize mechanical strength and functionality [10], as the manufacturing of these materials requires extensive steps, such as mold preparation, casting, and freeze-drying. However, from a surgeon’s perspective, materials that can be easily cut into the desired shape, such as foam, are preferable [10]. Additive manufacturing allows the three-dimensional (3D) printing of scaffolds with exact dimensions when surgery is in progress. These advancements in 3D printing have caused a paradigm shift in the use of composite materials for bone tissue engineering.

In this article, a perspective on the viability of polymer-bioactive glass composites as a mate-rial for the 3D printing of scaffolds for bone tissue engineering is provided. The next section is a brief overview of bioactive glass as a material and its mechanism inside a tissue environment. Next, we highlight the major additive manufacturing techniques that have been explored for 3D printing of bioactive glass composite scaffolds. Further, a critical perspective on different 3D-printed bioactive glass polymer composites for bone tissue engineering application is discussed.

## 2. Bioactive Glass: An Active Bone Regeneration Material

Bioactive materials are typically designed to undergo surface reactions when implanted inside the body. Bioactive glass forms a hydroxyapatite-like layer on its surface when implanted into the body, establishing a firm bond with bone tissues [10,34]. The first bioactive glass, created by Dr. Hench, was termed Bioglass^®^ 45S5 and had the composition of 45 wt.% ${\mathrm{SiO}}_{2}$, 24.5 wt.% CaO, 24.5 wt.% ${\mathrm{Na}}_{2}O$, and 6.0 wt.% $P_{2}O_{5}$ [46]. Since the invention of Bioglass^®^ 45S5, there have been numerous advancements in the field of bioactive glass, such as borate, borosilicate, and phosphate glasses [10,34,46].

With advancements in sol–gel chemistry, numerous compositions of bioactive glasses containing up to 90 mol.% ${\mathrm{SiO}}_{2}$ are now possible [10]. As the side effects of sustained dosage of antibiotics have been highlighted, therapeutic ions are being explored in bone tissue engineering for their osteogenic, angiogenic, and antibacterial capabilities in bone tissue engineering [47,48,49]. Researchers have investigated the use of bioactive glasses as a reservoir of ions, such as Ca, Mg, Cu, Zn, and Ag as well as rare-earth metals for therapeutic ions [50].

**Figure 1:**
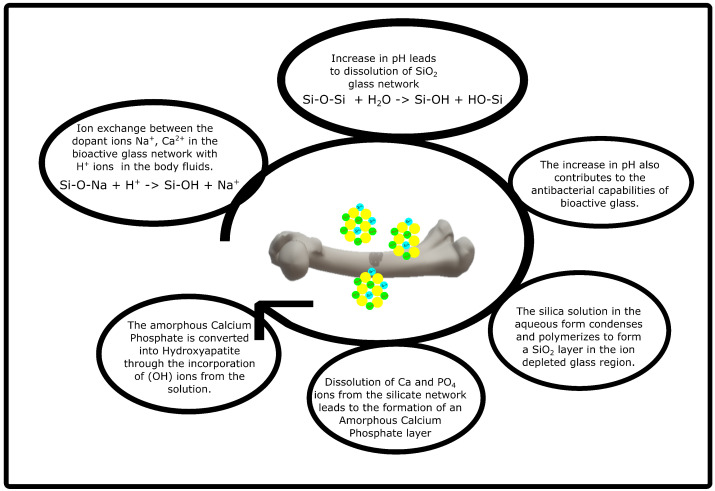
Schematic of the apatite formation mechanism in bioactive glass scaffolds.

For the development of new polymer/bioactive glass composites, the bone regeneration mechanism of bioactive glass needs to be studied. The mechanism of apatite layer formation has been widely understood using Stimulated Body Fluid (SBF); however, little is known about the mechanism at the interface between the apatite layer and bone tissue [10]. The steps of the mechanism of apatite formation can be explained in Figure 1 [10,46].

**Figure 2:**
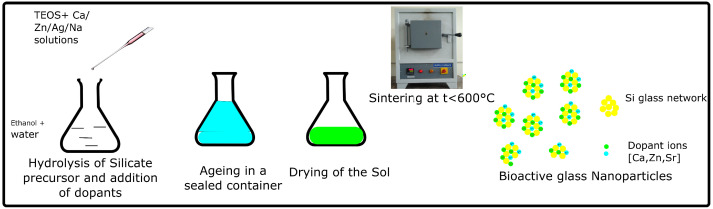
Schematic of the sol–gel synthesis of bioactive glass nanoparticles (image adapted from CC BY-SA 3.0 [52]).

Bioactive glasses were traditionally synthesized via a high-temperature melt-derived technique. The precursor materials $\left({\mathrm{SiO}}_{2},Na_{2}O,and\;P_{2}O_{5}\right)$ were mixed in a platinum crucible and then heated to approximately 1300 ${}^{\circ }$C. The glass particles were then crushed to obtain a fine powder of bioactive glass [51]. However, the particle sizes were in the micrometer range, making them unsuitable for extrusion-based 3D printing.Therefore, the sol–gel (Stöber) method, as shown in Figure 2, was developed to synthesize mesoporous nanoparticles of bioactive glass.

The sol-gel synthesis of bioactive glass can be performed at a temperature of 30 ${}^{\circ }$C. In sol-gel synthesis, the precursors, such as tetraethyl orthosilicate, are hydrolyzed and mixed with solutions of ions, such as Ca, Zn, Na, Ag, and Cu. The sol is then aged to form a gel and dried to evaporate the alcohol. The gel is then sintered at temperatures below 600 ${}^{\circ }$C to obtain bioactive glass nanoparticles. By adjusting on the pH of the hydrolyzing medium and the concentrations of the dopant ions and surfactants (such as ammonia solution or cetyltrimethylammonium bromide), it is possible to control the size of the particles obtained [51].

As surgeons prefer foams that can be cut during surgery, methods, such as thermal bonding [53,54] and polymer foam replication [55,56,57,58] have been used for the production of polymer foam bioactive glass scaffolds. Despite their promising biological properties, their mechanical properties were quite poor [53,54,55,56,57,58]. Using 3D printing, it is possible to fabricate customized scaffolds with good mechanical properties.

## 3. 3D Printing of Polymer/Bioactive Glass Composites: An Overview

Bone defects vary widely in dimension depending on the case. It is difficult for surgeons to determine the scaffold dimensions required unless the surgical procedure is initiated. This makes foam or cement the material of choice for surgeons. Surgeons prefer to cut the foam depending on the size required and insert them at the defect site [10]. However, foams lack the mechanical strength required for use as bone scaffolds.

**Figure 3:**
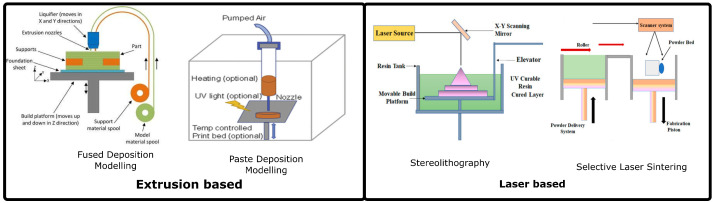
Additive manufacturing technologies used for the 3D printing of polymer-bioactive glass scaffolds. (Reproduced with permission from [61,62] under creative commons CC-BY license) (Reproduced with permission from [63] Copyright 2020, Elsevier).

With advancements in additive manufacturing technologies and imaging techniques, such as CT scans, it is now possible to design and print customized scaffolds [59]. Additive manufacturing or 3D printing is a manufacturing method adopted for the automated layer-by-layer fabrication of complex geometries using computer-generated models [60]. According to ASTM F2792 standards, there are seven families of 3D printing technologies, of which extrusion-based, or laser-based 3D printing is widely used for bone tissue engineering (Figure 3).

Selective laser sintering or selective laser melting is a laser-based additive manufacturing (AM) technique that uses a high-energy laser beam as the power source to sinter powdered material [64]. One of the main advantages of this process is the absence of a sintering step. After short cleaning and sterilization steps, the scaffold can be directly used in the implantation procedure. In a typical setup, bioactive glass is mixed with a polymeric binder and milled to obtain fine powder [65]. The powder is then hardened using a laser beam. Owing to the high temperature generated during the sintering process, the polymeric binding material is burned-out, and a pure glass scaffold is obtained. This makes selective laser sintering unsuitable for the fabrication of polymer-bioactive glass composite scaffolds.

In stereolithography-based 3D printing, the material to be printed is in the form of a photo-polymer resin. Using light from a light source, the area of interest is hardened through selective targeting. The bed is moved vertically, and the process continues layer-by-layer until a solid object was obtained. This technology allows the fabrication of complex geometries with a high accuracy for a wide range of materials. Polymers and ceramics have been successfully printed using this technology. A blend of photosensitive binder material with ceramic was used as feed for printing [64]. After the printing process is complete, the binder is removed through the debinding process, and the scaffold is finally sintered to obtain a dense glass scaffold.

Owing to the burnout of polymers in the high-temperature sintering process and concerns regarding the toxicity of photosensitive binders, extrusion-based additive manufacturing is the most preferred technology for the 3D printing of scaffolds. Fused deposition modeling is the most common 3D printing process. A typical setup involves using an extruder for heating a thermoplastic filament and depositing the molten plastic layer-upon-layer on a print bed until the final object is obtained [66,67].

The process is highly economical, and the results can be replicated easily and reliably using a low-cost 3D printer. If the technology is scaled up, polymer-bioactive glass composite filaments can be centrally produced and transported to different points of care. At the point of care, medical professionals can simply upload the CAD model and 3D print the scaffold with a simple click. Composite scaffolds can be used directly for implantation after a quick sterilization procedure.

Filaments of polymer-bioactive glass composites can be easily prepared using filament makers that are readily available in the market. In a typical filament-making process, bioactive glass particles are mixed with pellets of thermoplastic polymers. The mixture is passed through an extrusion setup consisting of a lead screw, multiple heating zones, and an extruder. The filament extruding from the extruder is wound into a spool using a motor.

Depending on the motor speed, the diameter of the extruded filament can be controlled. Paste deposition modeling (PDM), or direct ink writing, is an extrusion-based additive manufacturing process involving the deposition of a thixotropic paste on the print bed layer-upon-layer to fabricate solid objects [68]. PDM is used for 3D printing of liquid and non-thermoplastic polymers.

The polymers are dissolved in water or organic solvent to obtain a high-viscosity solution. Bioactive glass nanoparticles are then added to the liquid solution to prepare ink for printing [69,70]. The ink is subsequently loaded into the extruder and used to print the scaffold. After printing, the scaffold is heated to evaporate the solvent.The advantages and disadvantages of bioprinting through different 3D printing methods are summarized in Table 1.

## 4. Synthetic Polymers Used in Bioactive Glass Composites

Synthetic polymers are obtained synthetically using chemical precursors. The most common synthetic polymers are thermoplastics. Compared to naturally derived polymers, synthetic polymers are readily available because they are not derived from natural sources, have uniform properties, and allow greater scalability to fulfill production demands.

### 4.1. Thermoplastic Polymer/Bioactive Glass Composite

Thermoplastics are materials that soften upon heating above their glass transition temperature and then harden upon cooling. As of their excellent mechanical strength and 3D-printability, thermoplastics are the most preferred materials for the 3D printing of bioactive glass polymer composite scaffolds.

Various types of thermoplastic polymers, such as polylactic acid (PLA), poly(lactic-*co*-glycolic acid) (PLGA), polyvinyl alcohol (PVA), polycaprolactone (PCL), poly(hydroxybutyrate-*co*-hydroxyvalerate) (PHBV), and acrylonitrile butadiene styrene have been used for the fabrication of biomedical bone implants [60]. Among these polymers, PLGA, PCL, PLA, and acrylonitrile butadiene styrene-based implants have reached the clinical trial stage [71]. PLA, PLGA, and PCL have even received approval from the US FDA as materials for 3D printing of biomedical implants [72].

**Figure 4:**
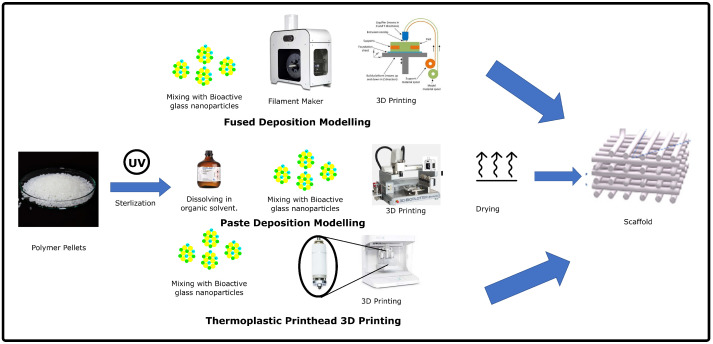
Workflow for different strategies for extrusion-based 3D printing of thermoplastic polymers-bioactive glass scaffolds. (Reproduced with permission from [61] under creative commons CC-BY license) (Reproduced with permission from [73] Copyright 2014, Elsevier).

Thermoplastics are suitable for 3D printing via both FDM and PDM. For FDM 3D printing of biofilaments, the polymer pellets are sieved and sterilized to eliminate contaminants that may affect scaffold cytotoxicity. The bioactive glass particles are then mixed with the pellets using a mixer. The mixture is then fed into a filament extruder to obtain a uniform printable filament. For PDM 3D printing, polymer pellets are dissolved in water or an organic solvent to prepare a paste. Bioactive glass nanoparticles are loaded into the paste and used for printing. After printing, the scaffold is dried to evaporate the solvent. In some modern 3D bioprinters, a thermoplastic polymer powder is loaded with bioactive glass nanoparticles and used directly to print the filament or prepare the paste. Figure 4 shows the different strategies for 3D printing of polymer-bioactive glass composites and the steps involved.

PLA is the most popular material for FDM 3D printing of polymer-bioactive glass compo-sites. It is a biodegradable material synthesized from corn starch [74,75]. Studies also inferred that the degradation products of PLA do not cause toxic reactions in the body [76,77]. In most studies, filaments were prepared by loading PLA with bioactive glass particles of up to 10 wt.%. The 3D-printed scaffolds showed good dimensional accuracy, mechanical strength, and bioactivity [78,79,80].

In several studies, stem cells were loaded with hydrogel printed with PLA-based ink to promote faster healing [79]. PLA bioactive glass scaffolds showed an increase in extracellular matrix (ECM) expression markers and a significantly higher VEGF-RNA value than the pure PLA scaffolds. The scaffolds were shown to induce higher proliferation of osteoblast cells compared with pure PLA scaffolds [79].

PCL is a biodegradable polymer with a melting point of 60 ${}^{\circ }$C and has a glass transition temperature of −60 ${}^{\circ }$C [81]. It has excellent mechanical properties, biocompatibility, and thermal stability. However, it is an inert material that does not actively promote bone formation. PCL-bioactive glass composite scaffold combines the mechanical and thermal properties of PCL with the osteogenic properties of bioactive glasses. Owing to its good rheology and viscoelastic properties, it is specifically suitable for PDM-based 3D printing of scaffolds. However, due to its insolubility in water, PCL inks were prepared by dissolving PCL in an organic solvents, such as chloroform or dichloromethane [82].

PCL was used for composite preparation with various bioactive glass compositions. The results of these studies also varied depending on the glass composition and particle size. Despite the significance of the improvement in bioactivity and cell proliferation, PCL scaffolds loaded with bioactive glass showed a significant reduction in mechanical properties compared to pure PCL scaffolds [82]. However, a recent study showed that 3D-printed PCL-bioactive glass composites exhibited mechanical properties and osteoconductive behavior similar to those of human trabecular bone. The results also revealed that the scaffolds were highly biocompatible [78].

**Figure 5:**
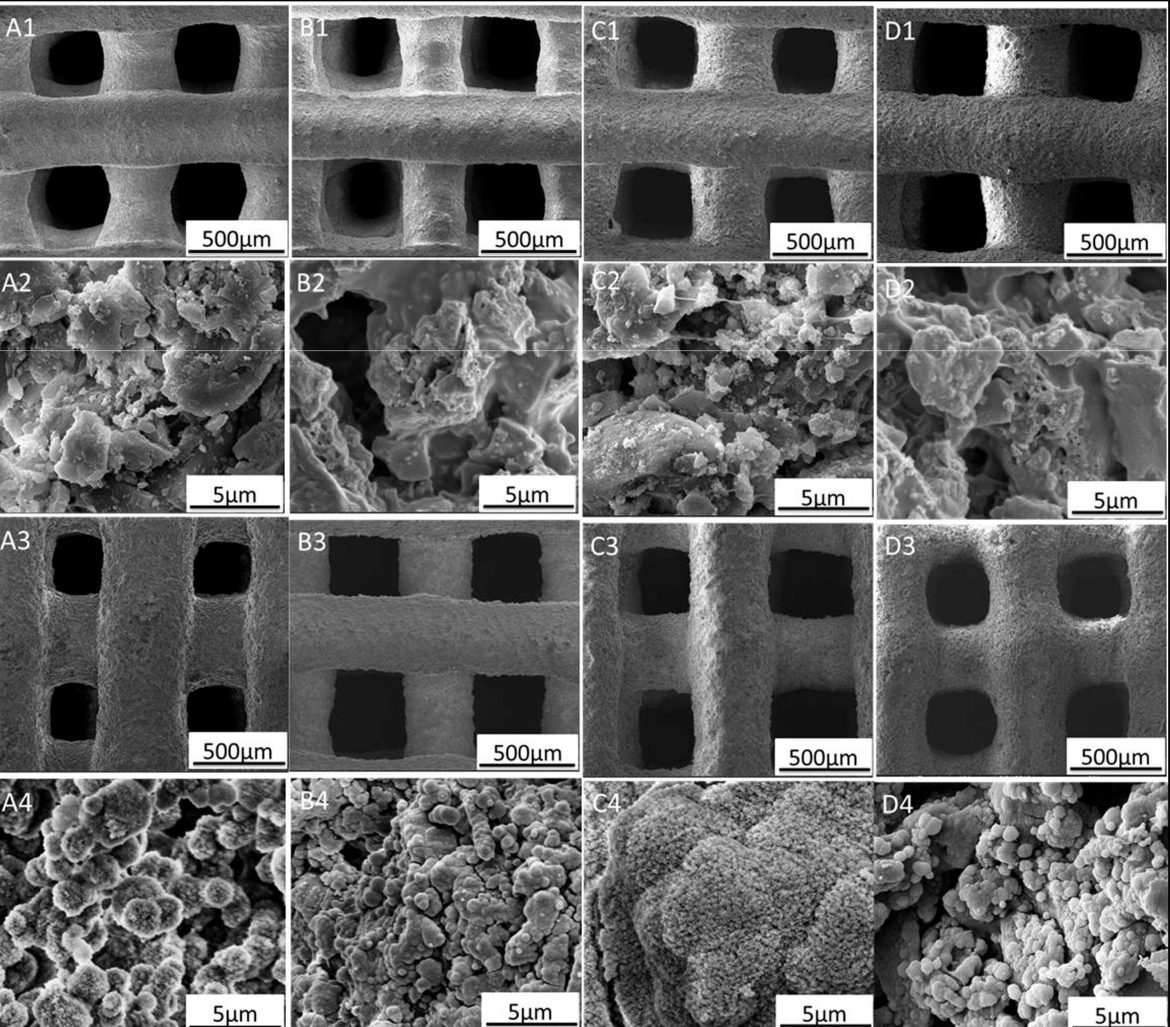
SEM morphology for PVA-Sr-containing bioactive glass scaffolds before and after 3 days of immersion in SBF: (**A1**,**A2**) MBG, (**B1**,**B2**) 5Sr-MBG, (**C1**,**C2**) 10Sr-MBG, and (**D1**,**D2**) 20Sr-MBG. Composition scaffolds before soaking in SBF: (**A3**,**A4**) MBG, (**B3**,**B4**) 5Sr-MBG, (**C3**,**C4**) 10Sr-MBG, and (**D3**,**D4**) 20Sr-MBG after soaking in SBF for 3 days. (Reproduced with permission from [73] Copyright 2014, Elsevier).

The mechanical properties and biocompatibility of PCL-BG composites were observed in another independent study [83]. Thus, PCL-bioactive glass composites could be potential candidates for bone tissue engineering. PVA is a water-soluble polymer used for both FDM and ink-based 3D printing. The main advantages of PVA are its biocompatibility and rapid solubility in water. A typical PVA was used as a binder for scaffold preparation. When the scaffold was implanted inside the body, the PVA rapidly dissolves, leaving a highly porous bioactive glass scaffolds (Figure 5). Owing to the rapid formation of hydrocarbon apatite layer on the scaffold structure, cell adhesion and proliferation were promoted [73,84].

Various other thermoplastic polymers have been used to 3D print polymer-bioactive glass composites. However, few studies have been conducted regarding the long-term biocompatibility and flexibility of these polymers. Owing to the lack of long-term studies, they are considered not interesting from the perspective of bone tissue engineering.

### 4.2. Thermoset Polymers/Bioactive Glass Composites

Thermoset polymers are mostly used in the form of resins for laser-based 3D printing. Poly(propylene fumarate) (PPF) is a photocrosslinking polymer. Although PPF does not show osteoconductivity or osteoinductivity, it is hypothesized that the addition of bioactive glass promotes its osteogenic properties [85]. In a previous study, 3D-printed PPF-bioactive glass composite scaffolds have been prepared using DLP 3D printing and studied for their bioactivity [85].

The results of the study appear promising; however, studies need to be conducted on cell viability to further ascertain its functionality. Poly(glycerol sebacate) (PGS) is a biocompatible and biodegradable polyester that has been explored for various tissue-engineering applications [86,87,88]. The material properties are programmed by controlling the curing time, curing temperature, and reactant concentration [86,89]. PGS is a soft elastomeric material whose application is limited to soft tissue engineering [87,88]. For hard-tissue engineering applications, PGS composites with bioactive glass have started gathering attention [87]. The scaffolds show negligible weight loss over long durations.

The scaffolds display superior mechanical properties, low cytotoxicity, high cell proliferation, and pH-balancing capabilities [90]. In one interesting study a PGS-PCL/bioactive glass scaffold was fabricated by combined 3D printing and electrospinning [91]. PGS-PCL mats are electrospun on a PGS-PCL/bioactive glass composite 3D-printed grid [91]. The scaffold displayed a pH-balancing system and superior mechanical properties compared to 3D-printed grids [91].

Biodegradable polyurethane (PU) is available in the form of foams. Due to the flexibility offered by the PU/bioactive glass scaffold, these will be the most favored choice of the surgeons. Studies on PU/bioactive glass composite scaffolds have shown a deformation of 350% and sufficient cell attachment and cell proliferation [92]. However, 3D-printed scaffolds have not been widely studied due to the challenges in optimizing the printing protocols for 3D printing of PU.

For drug delivery applications, bioactive glass was loaded with growth factors to promote healing process [93]. 3D printing of growth factor-loaded bioactive glass remains a challenge. Filament-based 3D printing requires a high temperature, and a laser-based process needs organic solvents [94,95,96]. These harsh environments hamper the functionality of the growth factors. To address this issue, novel printing techniques, such as light-mediated printing [97] and low-temperature printing [98] are being explored for 3D printing of bioceramic-polymer composites.

Table 2 lists some promising research works on thermoplastic polymer-bioactive glass com-posites. The results of these studies showed that the bioactivity and osteogenic capabilities of scaffolds were enhanced without a significant effect on their mechanical strength. However, the available data are limited on the comparison of polymer-bioactive glass scaffolds with pure bioactive glass scaffolds in terms of bioactivity, cytotoxicity, and osteogenic capabilities.

## 5. Natural Polymers Used for Bioactive Glass Composites

In recent years, advancements in tissue engineering have accelerated the development of biomimetic, biocompatible, nontoxic, and biodegradable biomaterials. Incorporating natural polymers in biomaterial fabrication is essential to retaining biological properties. Natural polymers can be classified into protein- and polysaccharide-based polymers [103]. Natural polymers combined with bioactive glasses have enhanced biological and mechanical properties [104]. Table 3 summarizes the combination of natural polymers with bioactive glass 3D-printed scaffolds for bone tissue engineering.

### 5.1. Protein-Based Polymer-Bioactive Glass Composites

Protein-based composites are widely used in bone tissue engineering. These proteins provide native ECM to bone cells. These proteins include collagen, gelatin, silk fibrin, and fibrin [103]. Collagen is one of the most abundant proteins in human tissues and an essential component of the bone. The composition of collagen in the bone is approximately 17–20%. In addition to providing the ECM, collagen plays a role in regulating cellular morphology, cell-cell interaction, cell adhesion, cell migration, and cell differentiation. Collagen assists osteoblasts in the mineralization process [110]. Although there are various advantages of using collagen for bone regeneration, the presence of collagen decreases the mechanical strength of a biomaterial.

Thus, it is preferable to use collagen combined with other polymers to increase the mechanical strength of 3D-printed scaffolds. Studies have been conducted using collagen, chitosan, and bioactive glass. This nanocomposite exhibited thermo-responsive behavior, gelating into a hard material at physiological pH, and thus can be used as an injectable material [111]. Silk fibroin is a protein extracted from cocoons and can be used to induce the osteogenic ability of cells and increase the mechanical strength of a scaffold when combined with mesoporous bioactive glass (MBG) [105].

**Figure 6:**
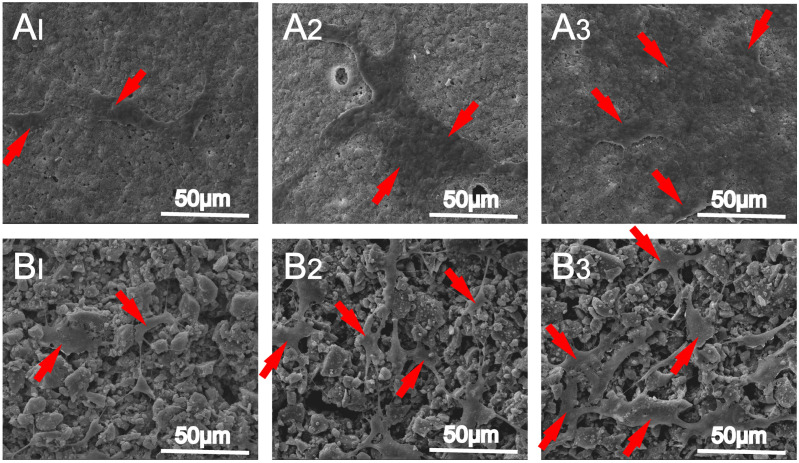
SEM images of hBMSCs attached on (**A**) MBG/SF and (**B**) MBG/PCL scaffolds for (**A1**,**B1**) 1, (**A2**,**B2**) 4, and (**A3**,**B3**) 7 days. The red arrows indicate cells. Reproduced from [105] under copyright CC BY licence.

Therefore, it is advisable to fabricate and cure silk fibroin-based scaffolds under mild and safe conditions. Mild conditions were maintained in a harsh chemical environment, and the helical structures of the proteins may transform into β-sheets, thereby, contributing to the instability of the composites. SF-MBG ink was 3D-printed via extrusion-based printing to obtain a scaffold (Figure 6). Properties of SF-MBG, such as the compression strength, porosity, and degradation rate, as well as its cellular properties, such as cell adhesion and proliferation, were more favorable than those of a PCL-MBG scaffold [105]. Thus, 3D-printed SF-MBG scaffolds could be promising biomaterials for bone tissue engineering.

Another method of 3D printing is to use a sacrificial layer that can provide the desired morphology in terms of the porosity of the scaffold [106]. This method is known as indirect 3D printing. This method integrates traditional and advanced tissue engineering aspects to provide improved and uniform micro- and macroporous structures to the scaffolds. This can be achieved by creating a template using removable plaster dies. After SF-BG is 3D printed onto the sacrificial mold, it is cured and subjected to boiling water to remove the sacrificial mold and obtain the SF-BG scaffold. Both methods of 3D printing of scaffolds showed excellent mechanical properties and biocompatibility and can therefore be considered potential candidates for bone tissue engineering applications [105,106].

### 5.2. Polysaccharide-Based Polymer-Bioactive Glass Composites

**Figure 7:**
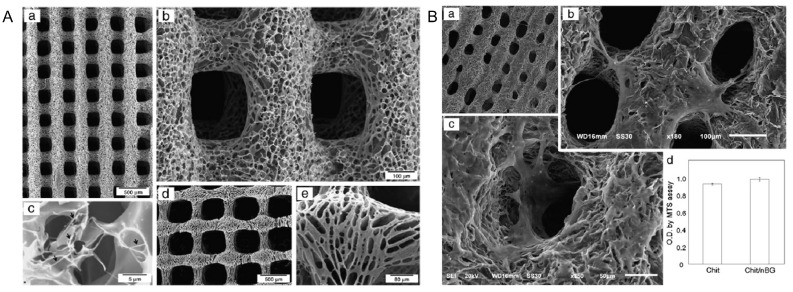
SEM morphologies of the robotic-dispensed scaffolds for bone tissue engineering. **A** (**a**–**c**) Chitosan/nanobioactive glass scaffolds and (**d**,**e**) Chitosan scaffold at different magnifications. **B** (**a**–**c**) Cells grown on chitosan/nanobioactive glass scaffolds for 3 days at different magnifications d Cell proliferative potential of chitosan and chitosan/nanobioactive glass scaffolds measured by MTS assay Reproduced from [107] with permission from Copyright 2011, Elsevier.

Chitosan is a positively charged polysaccharide; when combined with bioactive glass, it improves the mechanical and biological properties of the biomaterials. Chitosan is one of the most widely used biomaterials for bone tissue engineering because it also induces cell differentiation. In a previous study, bioactive nanoglass was dispersed in distilled water and mixed with chitosan solution. The ink was loaded into a syringe with a heating jacket maintained at 37 °C. The 3D-printed bone scaffold was deposited into a dry-ice cooling bath to allow solidification. The morphology of the scaffolds revealed the formation of aligned macrochanneled pore structures with diameters of approximately 10 µm (Figure 7A) [107].

It was observed that these scaffolds provided an optimum microenvironment for pre-osteoblastic cell growth, initiating cytoskeletal network formation (Figure 7B). Thus, 3D-printed chitosan-bioactive nanocomposite scaffold acts as a potential biomaterial for bone tissue engineering. However, the scaffold must be evaluated for osteoblast differentiation ability, and preclinical examination of these scaffolds would increase the rate of biodegradation and its role in bone regeneration.

**Figure 8:**
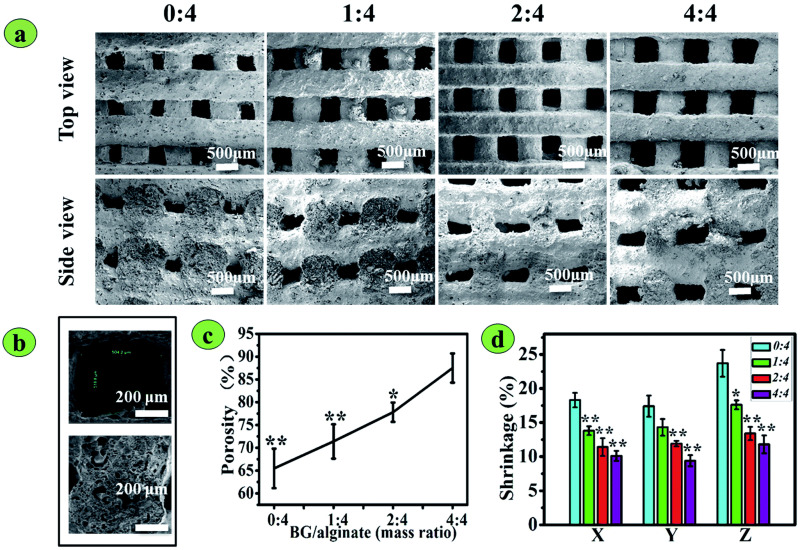
The morphology, porosity, and shrinkage of 3D-printed BG/SA composite scaffolds: (**a**) Top and side-view SEM images of the scaffolds. The BG/SA mass ratios were 0:4, 1:4, 2:4, and 4:4. (**b**) High- magnification SEM images showing a pore and a vertical section of strands of the BG/SA 2:4 scaffold. (**c**) Porosity. (**d**) Scaffold shrinkage (%) in the X, Y, and Z directions. Reproduced from [108] under copyright CC BY-NC 3.0.

Alginate is a natural anionic polymer originally found in seaweeds. Its hydrophilic, biodegradable, and gelling properties at physiological conditions have been widely used in scaffold engineering [112]. In a previous study, bioactive glass/sodium alginate (SA) composite scaffolds were 3D-printed and cured by incubation in calcium chloride solution [108]. The 13-93 BG/SA scaffold was optimized at different ratios, 0:4 (0% 13-93 BG), 1:4 (20%), 2:4 (33.3%) and 4:4 (50%) (Figure 8). These 3D-printed scaffolds were analyzed, and the 2:4 BG/SA composite was revealed as the most potent formulation. However, preclinical testing of these 3D-printed scaffolds is required to verify their capabilities.

**Figure 9:**
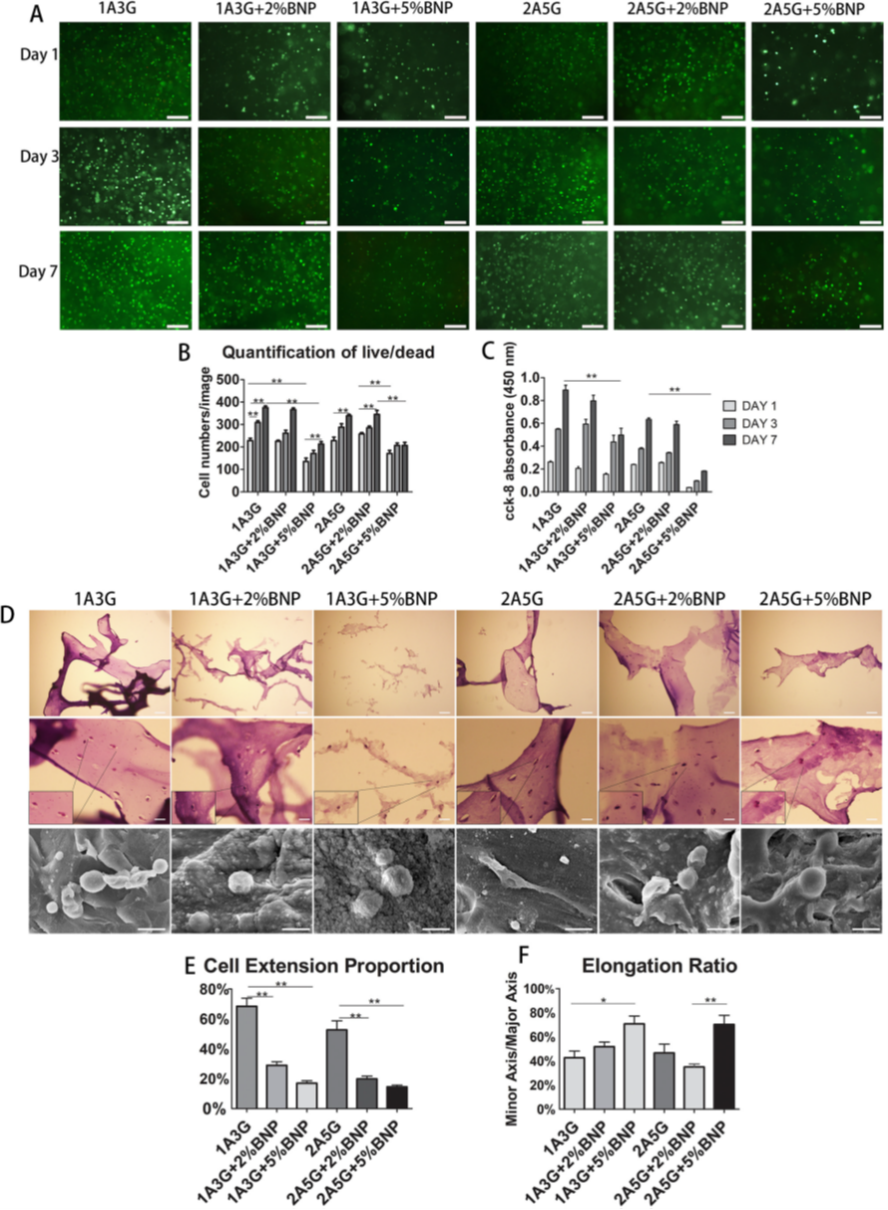
Cellular morphology of MSCs after treatment with different compositions of bioink. (**A**) Images representing live/dead cells in each bioink treatment groups at day 1, 3, and 7. (**B**) Quantitative analysis of the number of live cells. (**C**) Absorbance value at OD 450 nm in the CCK-8 cell proliferation assay. (**D**) Hematoxylin and eosin staining images depicting MSC spreading phenomenon. (**E**,**F**) Quantitative analysis of cellular extension and cell aspect ratio of extended cells, respectively. Reproduced from [109] with permission from Copyright 2019, Elsevier).

Alginate/gelatin hydrogel-based bioinks show good printability when combined with bioactive glass, making the composite a potential candidate for bone tissue engineering. The presence of alginate/gelatin can maintain the stemness of mesenchymal stem cells (MSCs). The composition of alginate-gelatin bioactive nanoparticles was optimized and sterilized before the addition of 2 × 10${}^{7}$ cells of MSCs/5 mL of the bioink. Hematoxylin and eosin staining of the scaffold was performed to analyze the structure and morphology of MSCs (Figure 9) [109].

## 6. Conclusions

3D-printed bioactive glass composite scaffolds have shown favorable results in preclinical studies, and it is believed that they have a great potential in clinics in the near future [44]. A major advantage of bioactive glass composites is their good mechanical properties and biological osteogenic properties. However, there is always a conflict between using natural polymers and synthetic polymers in combination with bioactive glass.

Natural polymers provide higher biocompatibility and biodegradability, whereas synthetic composites may provide scaffolds with higher mechanical strength [104]. Both polymers have advantages and disadvantages. However, the selection of polymers must be based on their application or the bone region that requires replacement. Thus, the design and optimization of the formulation plays a crucial role in the development of implants. Along with the formulation, the optimization of 3D-printing parameters and type of printing also play crucial roles in the maintenance of implants.

According to the literature survey, FDM- and PDM-based printings are two widely used methods for printing bioactive glass composites. However, FDM-based 3D printing showed greater printability and cost-effectiveness for implant development compared with PDM-based 3D printing [113]. Furthermore, these composites can be used to deliver drugs to target bone tissues in the future. Therefore, 3D-printed bioactive glass composites could be a potential target in the field of bone tissue engineering.

## Figures and Tables

**Table 1:** Advantages and disadvantages of different 3D printing technologies used for the fabrication of polymer-bioactive glass composite scaffolds.

Technology	Feedstock	Advantage(s)	Disadvantage(s)
Fused Deposition Modelling	Filament	Low cost High reproducibility.	Minimum resolution is 0.02 mm.
Paste Deposition modelling	Bioink/Paste	Resolution upto 1 µm.	Rheology of the bioink need to be tailored for efficient printing.Reproducibility depends on the rheology of bioink
Selective Laser Sintering	Granules of polymerand ceramic additives	Flexibility in geometries printed	Costly High temperature causes bioactive glass to lose amorphous nature.
Stereolithography	Vat of photopolymer inkand bioceramic	Resolution upto 170 nm	Toxicity of resins Exhaustive post processing steps are needed.

**Table 2:** Summary of results for studies in 3D-printed synthetic-polymer bioactive glass composites.

Polymer	Bioactive Glass Composition	Printing Technology	Summary of Results	Reference
PLA	45S5 45 ${\mathrm{SiO}}_{2}$–24.5 CaO–24.5 ${\mathrm{Na}}_{2}O$–6 $P_{2}O_{5}$ wt.%	FDM	Filaments prepared by mixing PLA and bioactive glass in ratios 0, 1, 2.5, 5 and 10 wt.% using a filament maker. Scaffolds loaded with 0–2.5% (wt) BG showed mechanical properties mimicking those of cancellous bone of human proximal tibias.	[78]
PLA	13-93B3 53% B_2_O_3_, 20% CaO, 12% K_2_O, 6% Na_2_O, 5% MgO, 4% $P_{2}O_{5}$	PDM	PLA BG scaffold is seeded with human adipose Stem cells. The mechanical properties of the scaffold improved in BG-loaded composites. The cell viability was non-uniform with the top layer showing higher viability and reduced viability at the bottom.	[79]
PLA	45S5 (45 ${\mathrm{SiO}}_{2}$–24.5 ${\mathrm{Na}}_{2}O$–24.5 CaO-6 $P_{2}O_{5}$ wt.%)	FDM	Confirmation of Hydrocarbonate Apatite layer formation when PLA/BG scaffold is immersed in Simulated Body Fluid.	[80]
PCL	45 ${\mathrm{SiO}}_{2}$–24.5 ${\mathrm{Na}}_{2}O$–24.5 CaO–6 $P_{2}O_{5}$ wt.%	DIW	PCL dissolved in dichloromethane and loaded with bioactive glass. Loading of BG reduces the mechanical strength of the scaffolds. BG-loaded scaffold showed significantly higher cell proliferation.	[82]
PCL	58S (mol%: 60${\mathrm{SiO}}_{2}$–36CaO–4$P_{2}O_{5}$	FDM	PCL Scaffolds studied with 0, 5, 10, 20 wt.% Bioactive glass. The higher bioactive glass composition enhances osteogenic differentiation. The higher the content of bioactive glass in the scaffold, the slower the degradation rate of the scaffold.	[99]
PCL	13-93B3	PDM	Dual extruder 3D printing of PCL/BG and Pluronic F127 hydrogel as cell suspension medium. The scaffolds show the formationof hdroxyapatite layer formation and excellent bioactivity.	[100]
PHBV	45S5	FDM	There is an increase in mechanical strength with increase in the infill. The biological and mechanical properties match that of ECM of trabecular bone	[101]
PCL	Cobalt and strontium doped Bioactive glass	PDM	The scaffolds displayed hydrophilicity, bioactivity, cell viability, and apatite formation capabilities.	[102]
PCL	Mg-containing bioactive glass	Precision Entrusion Deposition (FDM)	Different compositions were studied. Scaffold with 50:50 composition was found to be most suitable.	[33]
PVA	MBG powder (Si/Ca/P molar ratio 80/15/5)	PDM	Mesoporous bioactive glass scaffolds with hierarchical pore architecture. PVA is used as a binder for 3D printing of BG paste. The scaffolds showed impressive apatite formation capability and sustained drug-release capabilities.	[84]
PVA	Sr-MBG	PDM	Scaffold showed excellent mechanical strength, bone forming capabilities and bioactivity.	[73]
PPF	45S5	DLP	Resin was prepared for DLP technology and tested for bioactivity.	[85]
PGS-PCL	45S5	PDM+Electro-spinning	PGS-PCL mats are electrospun on PGS-PCL /bioactive glass composite 3D-printed grid. The scaffold displayed a pH-balancing system and superior mechanical properties compared to 3D-printed grids.	[91]

**Table 3:** Result summary of studies on 3D-printed natural polymer-bioactive glass composites.

Natural Polymer	Bioactive Glass Composition	Printing Technology	Summary of Results	Reference
Silk fibroin	Mesoporous BG NPs	PDM	Porosity and the compression strength of SF-MBG of higher than PCL-MBG BMP-2 and BSP expression was higher in SF-BG scaffolds	[105]
Silk fibroin	45S5	PDM	Compressive modulus and compressive strength of SF-µBG were superior to SF-nBG SF-nBG would provide a good environment for hBM-MSCs for growth and differentiation	[106]
Chitosan	$70\;{\mathrm{SiO}}_{2}\cdot 25\;\mathrm{CaO}\cdot$$5\;P_{2}O_{5}$ wt.%	PDM	Scaffolds showed higher cellular proliferation	[107]
Alginate	13-93	PDM	Porosity of these composites improved as the ratio of 13-93 BG increased in the scaffold	[108]
Alginate-gelatin	BG NPs	PDM	Bioactive glass scaffolds with 2:5 (SA:G) ratio showed needle-like MSC cells with membrane protrusions	[109]

## Data Availability

Data sharing is not available.

## Abbreviations

The following abbreviations are used in this manuscript:

3D	Three dimensional
µBG	micro bioactive glass
ALP	Alkaline phosphatase
BG	Bioactive glass
BMP-2	Bone morphogenetic protein-2
BNP	Bioactive nanoparticle
BSP	Bone sialoprotein
COL-1	Collagen-1
ECM	Extracellular matrix
G	Gelatin
hBM-MSCs	Human bone marrow-derived mesenchymal stem cells
MPa	Mega Pascal
nBG	nano bioactive glass
OCN	Osteocalcin
OPN	Osteopontin
PCL	Poly-caprolactone
rBM-MSCs	Rat bone marrow-derived mesenchymal stem cells
SA	Sodium alginate
SF	Silk fibroin
FDM	Fused Deposition Modelling
SLA	Stereolithography
PDM	Paste Deposition Modelling
